# Secondary Prevention of Osteoporosis: If Not Now, When?

**DOI:** 10.5041/RMMJ.10478

**Published:** 2022-07-31

**Authors:** Tzvi Dwolatzky

**Affiliations:** 1 Geriatric Unit, Rambam Health Care Campus, Haifa, Israel; 2 The Ruth and Bruce Rappaport Faculty of Medicine, Technion–Israel Institute of Technology, Haifa, Israel

**Keywords:** Fractures, older people, osteoporosis, secondary prevention

[Hillel] used to say: If I am not for myself, who will be for me? And if I am only for myself, what am I? *And if not now, when?*^1^

Global aging is associated with an increase in life expectancy and a rapid growth in the older population. The blessing of long life is often tainted by older people developing comorbidities and cognitive and physical frailty. Osteoporosis commonly develops as we age, yet many are not aware of the fact that they have osteoporosis until they suffer a fracture. While fracturing an osteoporotic bone frequently results from minimal trauma, the traumatic results of the fracture are enormous. The consequences of a fracture may include the loss of physical function and disability, delirium and cognitive impairment, an impaired quality of life, increased health costs, and the need for long-term care. All this is relevant to the first osteoporotic fracture, but each subsequent fracture places an even greater strain on the older patient and further challenges health care providers. Clearly, the optimal approach should be one of prevention. An emphasis should be placed on primary prevention, on achieving the highest peak bone mass possible by the promotion of a healthy lifestyle and adequate nutrition at a young age, by ensuring safe environments, and by screening for the early identification of osteoporosis for those who are at risk.

Once a fracture occurs, osteoporosis clearly reveals itself. We have made great advances in the surgical and rehabilitative care of osteoporotic fractures. Guidelines recommend undergoing surgery with minimal delay to prevent delirium, thrombosis, infections, and many other possible complications. Physiotherapy and early active mobilization have completely replaced the outdated strategy of prolonged bedrest. In-patient rehabilitative services are provided to encourage a return to the prior level of function as much as possible, and community and home-based rehabilitation services are developing and available in many countries. However, we tend to forget that recurrent falls are a common geriatric syndrome, and that when the older person with osteoporosis has fallen once, the subsequent fall is almost inevitable. In fact, about a quarter will fall again within a year, and half of these falls will occur within three months.^2^ Thus, a fall in an older person is a clear call for instituting an active approach in promoting secondary prevention.

The secondary prevention of osteoporosis has been clearly outlined in guidelines.^3^–^5^ Older people, particularly post-menopausal women, who present with a confirmed fracture occurring as the result of a low energy impact (such as a fall from standing height), should be provided a regimen of secondary prevention of osteoporosis. Dietary vitamin D and calcium supplementation should be provided. Treatment with a generic bisphosphonate should be initiated. The use of C-terminal telopeptide as a bone turnover marker is useful for monitoring the long-term efficacy of bisphosphonate therapy.^6^ For those who have persistent gastrointestinal symptoms and are intolerant to the generic bisphosphonate, or in the event of a fracture on treatment, particularly where an increase in C-terminal telopeptide is demonstrated, a referral to a specialist osteoporosis clinic is usually justified. In this case, other bisphosphonates and therapeutic options should be considered.

While there are some differences among the various guidelines, there is a consensus that such an approach should be initiated following a likely osteoporotic fracture. However, at a practical level, such guidelines are frequently not followed. Many studies have reported that there is disregard for the initiation of secondary prevention strategies post-fracture. In the study published in this July issue of *Rambam Maimonides Medical Journal*, Aypak et al. performed a cross-sectional study reviewing the medical records of 214 patients with osteoporotic fractures.^7^ As expected, the majority of patients (65.7%) were not taking osteoporosis-related drugs prior to the fracture. Post-fracture, almost two-thirds of the patients commenced treatment with anti-osteoporotic drugs, but this decreased to only 41.3% after a year. The authors concluded that the use of drugs for secondary prevention is insufficient and that many patients discontinue treatment over a relatively short time. In fact, these findings show higher rates of adherence to guidelines than previous studies.

It is generally reported that only about 20% of those with a fracture will subsequently receive treatment for osteoporosis. A prospective observational study conducted at eight different trauma centers in Austria as part of the International Costs and Utilities Related to Osteoporotic Fractures Study (ICUROS) enrolled 915 patients (78% female). Only 20% were receiving osteoporosis treatment at the time of fracture. For those women with no osteoporosis treatment at the time of the index fracture, only 17.6% were receiving treatment at 4 months, and this declined to 15.3% at 18 months. For men these rates were lower. The most frequently prescribed drugs were bisphosphonates (88%). About one-third of the subjects were receiving supplementation with calcium and vitamin D at the time of fracture, and for those without prior supplementation less than half of the women and a quarter of the men were still taking supplements at 18 months.^8^

Many factors influence the rate of initiation of drug treatment for secondary prevention of osteoporosis. An important factor is that of timing. It is reasonable to assume that the acute and traumatic event of a fracture and the exposure to health care teams that are actively involved in the care of patients suffering complications from osteoporosis should constitute a fertile ground for initiating treatment preventing further fractures. A follow-up study compared patients with fragility fractures at two medical centers. One center initiated in-hospital pharmacologic therapy for osteoporosis, while the other center recommended delayed post-hospital care by the primary care physician. At baseline none of the participants was receiving treatment for osteoporosis. At 6-month follow-up, 67% of those who had initiated therapy during hospitalization were still receiving treatment, while only 30% of patients in the delayed-care group were receiving osteoporosis-related therapy.^9^ Another study showed a clear advantage to the initiation of bisphosphonates within 3 months following fracture.^10^ While the long-term benefits are clear, there are those who question the benefit of early initiation of therapy on fractures, showing that they may not necessarily improve the rates of fracture non-union or malunion.^11^

Osteoporosis treatment after hip fracture was discussed previously in a well-received commentary.^12^ Variations among countries and health care systems are great, and there are reports of a decrease in drug treatment for the secondary prevention of osteoporosis. For example, evidence from a large US population study found a marked decrease in the use of effective drug treatment of osteoporosis in the 180 days following hip fracture, from 9.8% in 2004 to 3.3% in 2015.^13^ This is surprising, since the benefits of oral or parenteral bisphosphonate therapy on preventing subsequent femoral or vertebral fracture have been clearly shown. A recent meta-analysis investigated the efficacies of the five most used bisphosphonates for the secondary prevention of osteoporotic fractures.^14^ Overall, bisphosphonate use significantly reduced the risk of new vertebral (OR 0.56), hip (OR 0.69), and non-vertebral non-hip (OR 0.82) fractures. In this analysis, alendronate was the best intervention, with the lowest incidence of vertebral fractures (14.6%) and new hip fractures (18.5%) compared to four other drugs of this class.

It is important to emphasize that mortality following hip fractures in the older population is high, with reports of 1-year mortality in the range of 24%–33%.^15^,^16^ One-year mortality following osteoporotic vertebral fractures is also high (6.7%–28%),^17^ and a recent study reported that bisphosphonate use significantly lowered mortality in osteoporotic vertebral fractures.^18^ However, there is some concern regarding the long-term use of bisphosphonates, particularly regarding the appearance of atypical femur fractures. In a large population-based study the risk of atypical fracture increased with longer duration of bisphosphonate use.^19^ The study also found that bisphosphonate discontinuation was associated with a rapid decrease in the risk of atypical fracture. Putting their findings into perspective, the authors emphasize that the absolute risk of atypical femur fracture nevertheless remained very low as compared to the risk-reduction benefit of hip and other osteoporotic fractures. However, concerns regarding the use of bisphosphonates have resulted in the increased use of other classes of drugs for the secondary prevention of osteoporosis.^5^,^20^

Even putting the personal benefits on the health and well-being of the individual patient aside, health care policymakers should surely consider promoting an active program for the secondary prevention of osteoporotic fractures. Such an approach will help to prevent the significant costs involved in acute care and surgery, rehabilitation, and long-term care of patients with subsequent fractures.

While the challenge is great, obstacles must be recognized and addressed. Probably one of the greatest obstacles is a lack of awareness. Medical teams at the forefront of the acute care of patients with osteoporotic fractures should receive focused education regarding the implementation of guidelines for the secondary prevention of osteoporosis. The availability of a metabolic bone unit consultant service or a fracture liaison service in the orthopedic unit has been shown to be useful in some centers. Continuity of care with timely transfer of medical data to the primary care physician is also essential. Primary care physicians should be made aware of local guidelines for the secondary prevention of osteoporosis and should be provided support from community specialists or osteoporosis clinics. Adverse events should be identified, and the choice of drug adjusted accordingly in order to promote better patient compliance. Follow-up efforts must be made to encourage patients to continue treatment over an extended period of time. Health care costs in providing secondary prevention for osteoporosis should be subsidized and affordable.

The time has come for us to work together in a concerted effort to decrease the related suffering and consequences of osteoporotic fractures. *And if not now, when?*
